# MRI investigation of glymphatic responses to Gd‐DTPA infusion rates

**DOI:** 10.1002/jnr.24325

**Published:** 2018-09-11

**Authors:** Guangliang Ding, Michael Chopp, Lian Li, Li Zhang, Esmaeil Davoodi‐Bojd, Qingjiang Li, Zhenggang Zhang, Quan Jiang

**Affiliations:** ^1^ Departments of Neurology Henry Ford Hospital Detroit Michigan; ^2^ Department of Physics Oakland University Rochester Michigan

**Keywords:** cerebrospinal fluid, glymphatic system, infusion rate, magnetic resonance imaging, rat brain, RRID:SCR_007037

## Abstract

The glymphatic system is a newly identified waste clearance pathway in brain discovered and investigated predominately using in vivo two‐photon confocal microscopy. Magnetic resonance imaging (MRI), in contrast to two‐photon confocal microscopy, provides dynamic and real‐time pictures of the glymphatic system in whole brain. We employ MRI to investigate the response of the glymphatic system to the rate of infusion of Gd‐DTPA (magnevist). Wistar rats were subjected to a surgery of inserting a tube into the cisterna magna for infusion during MRI. Three infusion rates were chosen for 20 min infusions of diluted magnevist into the cerebrospinal fluid (CSF) of rat brain. Glymphatic response was imaged using dynamic MRI 3D measurement for 5 hr. Robust correlations were found in all ventricles between the peak intensities of image enhancement and infusion rates, with additional correlations between the peak times of MRI image enhancement and infusion rates in the fourth ventricle. An infusion rate of 2.92 μL/min induced an evident accumulation of tracer in the fourth ventricle near the cisterna magna. In hippocampal tissue, image enhancements exhibited low correlation with the infusion rates. However, an infusion rate of 1.67 μL/min provided a high image enhancement, but less tracer accumulation near the cisterna magna. Contrast‐enhanced MRI provides a suitable tool for investigating image contrast infusion rate response of the glymphatic system in rat brain. Considering both *T_1_* and T_2_^*^ effects in response to the infused magnevist into CSF, the infusion rate of 1.67 μL/min appears suitable for MRI study of the glymphatic system in rat.

**Significance**

The recently discovered glymphatic system, a waste clearance pathway in brain, may alter and improve our knowledge on the brain clearance system. MRI can provide quantitative evaluation of the whole glymphatic system. We found that infusion rate of 1.67 μL/min was suitable in MRI study of the glymphatic system in rat. Glymphatic function is depressed by brain injury and diseases, such as traumatic brain injury, stroke, Alzheimer’s disease, and diabetes. Thus, our results can be employed to investigate efficiency of the impaired glymphatic system with brain diseases, which may provide a new way to early diagnose and possibly treat neurodegenerative diseases.

## 1 INTRODUCTION

The glymphatic system is a recently discovered waste clearance pathway in brain (Iliff et al., 2012). Contrary to the traditional model of cerebrospinal fluid (CSF) reabsorption, a large proportion of subarachnoid CSF recirculates through the brain parenchyma along para‐vascular spaces and exchanges with the interstitial fluid (ISF), which is now called the glymphatic pathway, or glymphatic system (Iliff et al., 2012). In the glymphatic system, subarachnoid CSF rapidly enters the brain parenchyma along para‐arterial channels surrounding penetrating arteries to exchange with ISF, which is in turn cleared from the brain parenchyma along para‐venous pathways surrounding draining veins (Bedussi, Wel, et al., 2017; Iliff et al., 2012). The flow of fluid along these para‐vascular routes and through the interstitium is supported by transglial water movement through astrocytic aquaporin‐4 water channels and facilitates the efficient clearance of interstitial solutes from the brain parenchyma (Iliff et al., 2013, 2012 ). CSF movement along these para‐vascular pathways is driven by arterial pulsation, as well as with the respiratory pulsation (Bedussi, Almasian, de Vos, VanBavel, & Bakker, 2017; Iliff et al., 2013; Kiviniemi et al., 2016; Rennels, Gregory, Blaumanis, Fujimoto, & Grady, 1985).

This new waste clearance pathway may alter and improve our knowledge on the brain clearance system in at least two aspects. (Xie et al., 2013). The first and traditional point of view about the central nervous system (CNS) waste clearance is that, the CNS does not have histologically identifiable classical lymphatic vessels, and thus the CNS lacks the discrete pathways for interstitial solute and fluid clearance present in other peripheral tissues. (Kipnis, Gadani, & Derecki, 2012; Pardridge, 2011; Ransohoff & Engelhardt, 2012). A second prevailing interpretation has been that the CSF flows into the subarachnoid space surrounding the brain, and from here exits the cranial cavity by outflow along cranial and spinal nerves, the olfactory bulb and the arachnoid villi, which means the major part of the aqueous content of CSF directly drains into the venous sinuses via arachnoid granulations. (Boulton et al., 1996; Tripathi, 1974). These studies demonstrated that CSF circulation around blood vessels penetrating from the subarachnoid space into the Virchow Robin spaces provides both a drainage pathway for the clearance of waste molecules from the brain and a site for the interaction of the systemic immune system with that of the brain. (Brinker, Stopa, Morrison, & Klinge, 2014). The flow dynamics of CSF are thought to be altered in many pathological conditions. As a result, the clearance rate of brain waste via the glymphatic system may be altered or impaired by multiple physiological conditions and neurodegenerative diseases. Increase in 60% in efficiency of CSF‐ISF exchange had been demonstrated in sleeping brain compared to awake brain in mouse because of expansion and contraction of the extracellular space. (Xie et al., 2013). With an injection or infusion via the cisterna magna of magnevist^®^ (Bayer Inc., Whippany, New Jersey, US), a widely used clinical image contrast agent of Gd‐DTPA (gadolinium‐diethylenetriamine penta‐acetic acid), dynamic magnetic resonance imaging (MRI) of whole brain in rats shows that the CSF can be quickly recycled into the brain and exchanged with ISF. (Iliff et al., 2013). In addition, the glymphatic system in rats with type 2 diabetes mellitus (T2DM) is significantly impaired as evidenced by a reduced rate of removal of magnevist particles from the brain parenchyma, compared with non‐T2DM rats (Jiang et al., 2016). These results suggest that the clearance rate of glymphatic system is a sensitive index or biomarker of neurodegenerative diseases.

Interestingly, recent studies have also demonstrated that functional meningeal lymphatic vessels lining the dural sinuses are able to carry both fluid and immune cells from the CSF, and are connected to the deep cervical lymph nodes in both animals and humans, (Absinta et al., 2017; Aspelund et al., 2015; Louveau et al., 2015) which suggest that the lymphatic system participates and cooperates with the CSF to clean interstitial waste out of brain. However, the relationships of the newly discovered meningeal lymphatic vessels in dura, the glymphatic system in brain parenchyma, as well as the olfactory and cervical lymphatics are unclear and additional investigations are warranted (Engelhardt et al., 2016; Louveau et al., 2017).

MRI, with image contrast, is a powerful and competitive means to investigate the glymphatic system. MRI provides dynamic real‐time images of whole brain which facilitates investigation of influx and efflux processes of the glymphatic system. Hence, with processing and analysis, MRI can provide quantitative evaluation of the whole glymphatic system. MRI studies will allow investigation of whether a higher infusion rate will result in more contrast agent in glymphatic pathways, and thereby provide a temporal clearance curve for quantitative evaluation of the glymphatic system. On the other hand, rapid infusion of fluid into the CSF may interfere with the normal CSF volume and flow, (Bedussi, Wel, et al., 2017) which may alter the glymphatic system and hence influence the reliability of measurements. To date, there have been no MRI studies to address the glymphatic system response to infusion rate. We, therefore, performed the present MRI study to investigate the effects of infusion rate on glymphatic responses, which may increase our ability to obtain reliable and quantitative evaluations of the glymphatic system in rat brain.

## 2 MATERIALS AND METHODS

All experimental procedures were conducted in accordance with the National Institutes of Health (NIH) Guide for the Care and Use of Laboratory Animals and approved by the Institutional Animal Care and Use Committee of Henry Ford Health System.

### 2.1 Animal model and experimental protocol

**Figure 1:**
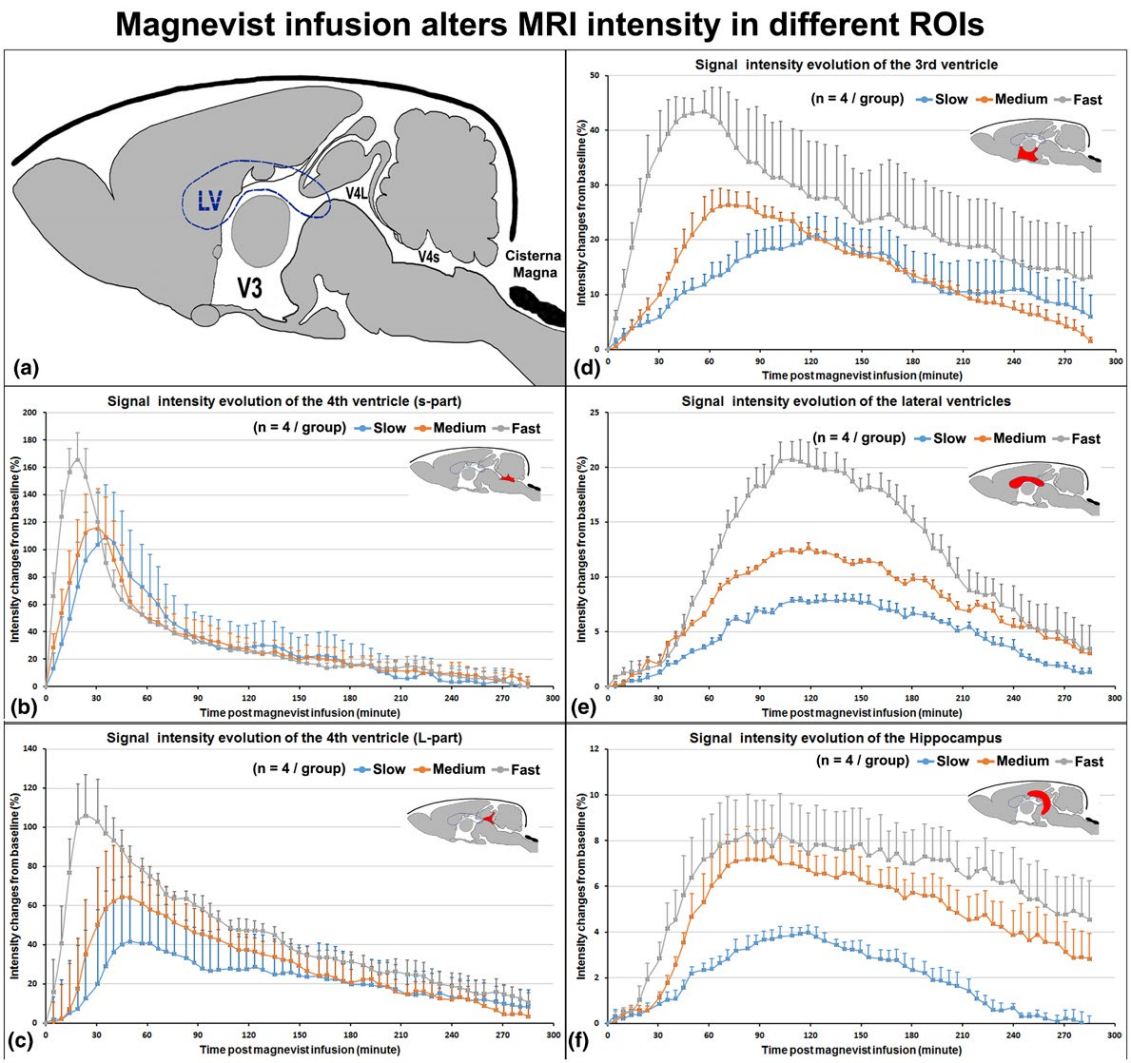
A sketch of ventricles of rat brain, showing their relative positions to cisterna magna (a). Evolutions of MRI image enhancement in V4s, the smaller cavity part of the fourth ventricle and the closest ROI to cisterna magna, were measured before and after magnevist infusion (b), where the enhancements reached peaks of approximately 165%, 115%, and 109% beyond baseline at times of 19, 31, and 36 min after onset of infusion, respectively, coinciding with the magnevist infusion rates of 2.92, 1.67, and 1.17 μL/min. For ROI of V4L, the larger part of the fourth ventricle, the same measurements were 106% versus 24 min, 64% versus 45 min and 42% versus 50 min (c). As to ROIs of the third ventricle (V3) and the lateral ventricles (LVs), these values were measured as 43% versus 57 min, 26% versus 76 min and 21% versus 124 min (d); as well as 21% versus 109 min, 13% versus 119 min and 8% versus 145 min (e). Hippocampus was the only ROI rather than ventricles here. The corresponding measurements were 8.3% versus 83 min, 7.3% versus 97 min, and 4.0% versus 119 min (f), respectively

Young healthy adult (175–200 g, 2–3 months) male Wistar rats (Charles River, Wilmington, MA, US) were used in the present study. A total of 12 rats were subjected to a surgery of inserting a PE10 polyethylene tubing (catalog number: 427,401; Becton Dickinson, MD, US), which was approximately 10 μL in volume filled with saline, into the cisterna magna of rat brain, as sketched in Figure 1a. Briefly, the head of the anesthetized rat was mounted in a stereotactic frame and the skin and muscle at midline of dorsal neck were cut to expose the occipital bone. Using a MicroMotor (Foredom Electric Co., Bethel, CT, USA), a 1 mm diameter hole through the skull was drilled approximately 1 mm above the cisterna magna and laterally 1 mm away from the midline of the skull. After opening the dura mater and arachnoid with a 27‐gauge needle, an approximately 2 mm long segment of the tube was advanced into the intrathecal space, and then the hole was sealed and the outside tube was fixed onto the occipital bone with superglue. Finally, the skin and muscle incisions were sutured closed. Animals were randomly assigned into three groups (4 rats per group) with different infusion rates for each group. The PE10 polyethylene tubing was fixed to the skull using superglue. The saline diluted magnevist (21.7 mM) was loaded into a PE10 catheter extension connected with the indwelling catheter to extend out of the magnet, and ready for infusion. The CSF volume is approximately 90 μL in rat brain (Pardridge, 2011) and the CSF production rate is between 2.66 and 2.84 μL/min (Chiu et al., 2012). In the present MRI study, referring to the approximate 2.75 μL/min (median of 2.66–2.84 μL/min)CSF production rate in rat, (Chiu et al., 2012) three rates of 1.17 μL/min (referred as slow infusion in present study), 1.67 μL/min (referred as medium infusion) and 2.92 μL/min (referred as fast infusion) were chosen for infusion of magnevist into CSF via the cisterna magna in the same total infusion duration of 20 min using a 100 μL syringe (Hamilton Robotics, Reno, NV, US) with an infusion pump (Harvard Apparatus, Holliston, MA, US), leading to total infusion doses of 23.3, 33.3, and 58.3 μL, respectively.

### 2.2 MRI measurements

MRI measurements were performed with a 7 T system (Bruker‐Biospin, Billerica, MA, US). A birdcage type coil was used as the transmitter and a quardrature half‐volume coil as the receiver. During MRI measurements, breathing of animals was monitored (Biopac Systems Inc., Goleta, CA, USA) and anesthesia was maintained using a gas mixture of N_2_O (70%) and O_2_ (30%) with 1.0%–1.5% isoflurane (Piramal Inc., Bethiehem, PA, US). Stereotactic ear bars were used to minimize head movement during the MRI scan for all rats, and rectal temperature of animals was maintained at 37 ± 1.0°C using a feedback controlled air heating blower (Rapid Electric, Brewster, NY, US).

Rats were placed prone in a cradle which was pushed into the magnet and positioned to the center inside magnet at the beginning of MRI scan using a fast gradient echo imaging sequence which produces three images of orthogonal slices. The dynamic magnevist influx and clean‐out process was monitored using a three dimension (3D) gradient echo imaging sequence with echo time of 4 ms, repetition time of 15 ms, flip angle of 15°, 32 × 32 × 16 mm^3^ field‐of‐view in coronal orientation, and 256 × 192×96 matrix, which yields an original image resolution of 125 µm × 167 µm × 167 µm. The bandwidth is 195 Hz/pixel. The individual scan time of 3D sequence was 4 min 37 s and continuously run for 5 hr with three baseline scans followed by the intra‐cisterna magna infusion of the diluted magnevist while MRI acquisitions continued (Iliff et al., 2013). The short term frequency drift rate of scanner is 0.59 Hz/hr, and the maximum frequency drift in 5 hr were equivalent to 1.5% of voxel in any one dimension.

### 2.3 Data and statistical analysis

The MRI 3D images were interpolated to 256 × 256×128 pixels, leading to a 125 µm ×125 µm ×125 µm spatial resolution for analysis. To correct for head motion, the general MRI processing procedure of registration was employed using *Statistical Parametric Mapping version 8* (SPM8, https://www.fil.ion.ucl.ac.uk/spm/; RRID: SCR_007037) (Jiang et al., 2016). Briefly, the first step is to extract out brain images by removing non‐brain tissue to reduce the computations and improve the performance of following registration steps. The second, using rigid body alignment of each scan to the time‐averaged (mean) image, to correct scan‐to‐scan mis‐registration caused by head movement. And the third, to ensure that voxel intensity represents percentage change relative to the average baseline images, all‐time series images were subtracted and divided by the baseline average image.

The averaged baseline images as well as the contrast‐enhanced MRI images were used to anatomically guide placement of each region‐of‐interest (ROI). The mean values of image intensities in preselected anatomical areas were measured on the registered 3D images over time, which produced time evolution curve of image intensity changes in every specific ROI, due to the uptake and cleanout of the paramagnetic contrast agent by regional brain tissue. Each measured temporal evolution curves was smoothed by an one dimensional low‐pass filter with the mask array of ($\frac{1}{16}$, $\frac{3}{16}$, $\frac{8}{16}$, $\frac{3}{16}$, $\frac{1}{16}$) in time domain, that is, the *n*th intensity I*_n_* = $\frac{1}{16}$I*_n__‐2_* + $\frac{3}{16}$I*_n‐1_* + $\frac{8}{16}$I*_n_*+ $\frac{3}{16}$I*_n__+1_* + $\frac{1}{16}$I*_n__+2_*, and was afterwards plotted for demonstration and further analysis.

Data analysis was performed in a blind fashion. No assumptions were tested in the present study, and the measurements were non‐repeated at the single highest level. MRI measurements are summarized as mean and standard deviation. Linear regression using the least squares fitting and Pearson's correlation analysis were applied for MRI measurements.

## 3 RESULTS

**Figure 2:**
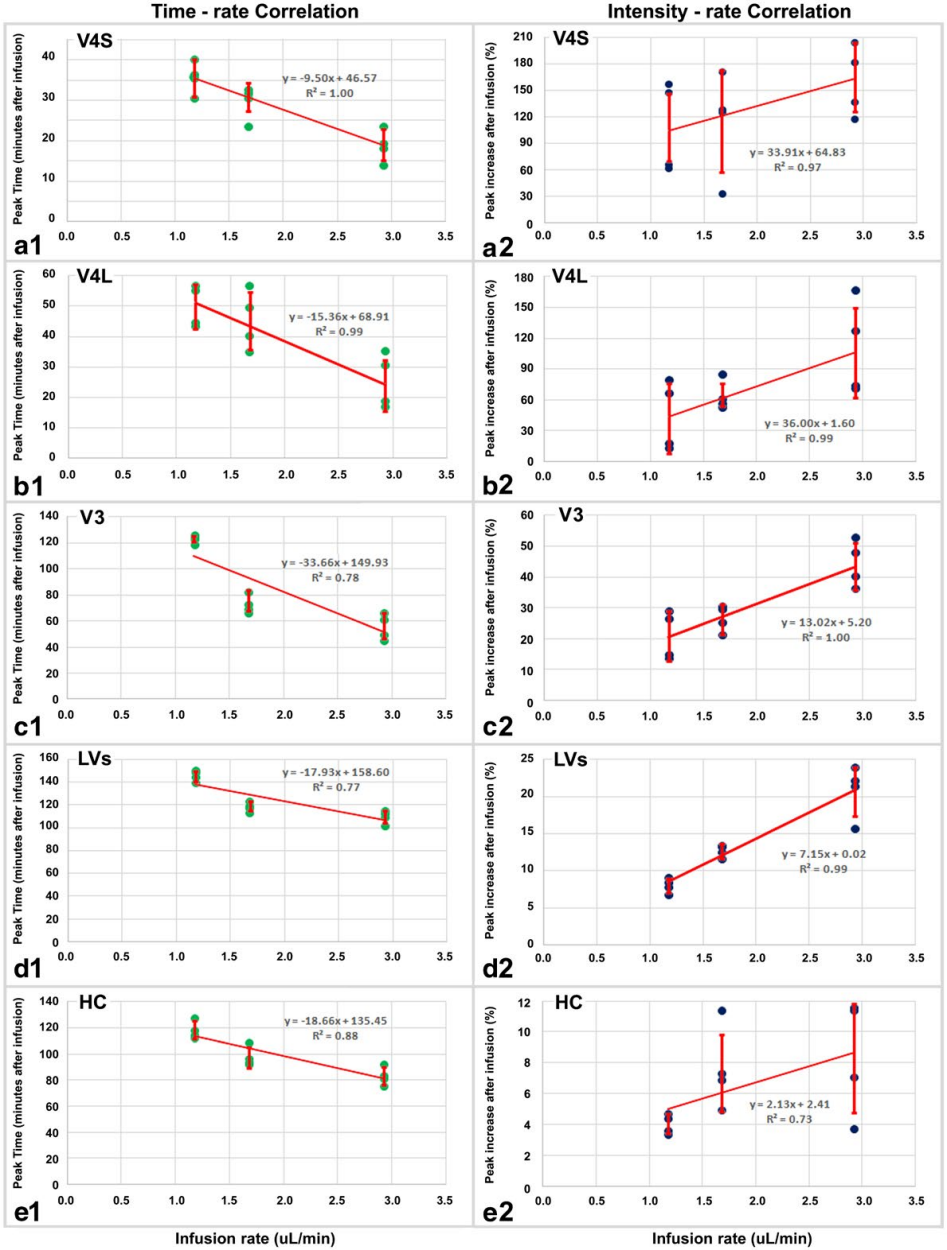
Analyses were displayed in the first column (left) to demonstrate the correlations of the peak times of MRI image enhancement with the infusion rates. In the order of V4s (a1), V4L (b1), V3 (c1), LVs (d1) and hippocampus (e1), the values were R^2^ = 1.00, R^2^ = 0.99, R^2^ = 0.78, R^2^ = 0.77 and R^2^ = 0.88. The second column (right) exhibited correlations between the increased peak intensities of MRI image (percentages referred to baseline) and the infusion rates, in the same order, the values were R^2^ = 0.97 (a2), R^2^ = 0.99 (b2), R^2^ = 1.00 (c2), R^2^ = 0.99 (d2) and R^2^ = 0.73 (e2). Bars are 95% confidence intervals

Figure 1a is a sketch of ventricles of rat brain, showing their relative positions to the cisterna magna, where the imaging contrast agent was infused into brain. The V4s, as the smaller cavity part of the fourth ventricle, was the closest ROI in distance to the cisterna magna for our MRI measurements. The temporal profile of MRI signals after magnevist infusion was measured and is drawn in Figure 1b. After the onset of infusion, MRI signal increased and reached approximate peak amplitudes of 165%, 115%, and 109% beyond the baseline at times of 19, 31, and 36 min, respectively, coinciding with the infusion rates of contrast agent at 2.92, 1.67, and 1.17 μL/min, as shown in Figures 1b, 2a1, and 2a2. The analysis demonstrated that the correlation between the peak times and infusion rates was R^2^ = 1.00, while it was R^2^ = 0.97 between the peak amplitudes and infusion rates. ROI of V4L is the larger part of the fourth ventricle, and the peak times of MRI signal elevation were measured as 24, 45, and 50 min after infusion onset (Figure 1c), which correlated with infusion rates from fast to slow at R^2^ = 0.99, shown in Figure 2b1. The image intensity increases were 106%, 64%, and 42% over the baseline at peak, and resulted in a correlation coefficient of R^2^ = 0.99 with the infusion rates from fast to slow infusion (Figure 2b2).

The same measurements were applied to the ROIs of the third ventricle (V3) and the lateral ventricles (LVs), as shown in Figure 1d and 1e, respectively. For the V3, the peak times were 57, 76 and 124 min with a correlation coefficient *R* ^2^ = 0.78 with infusion rates of 2.92, 1.67, and 1.17 μL/min (Figure 2c1); and peak increases of MRI intensity were 43%, 26%, and 21% of baseline with a correlation coefficient *R* ^2^ = 1.00 (Figure 2c2). As to the LVs, the corresponding measurements were 109, 119, and 145 min with a correlation coefficient of *R* ^2^ = 0.77 for the peak times (Figure 2d1), and 21%, 13%, and 8% with a correlation coefficient of *R* ^2^ = 0.99 for the peak intensities (Figure 2d2).

The hippocampus plays an important role in spatial navigation and long‐term memory, and may also play an important role in neurodegenerative diseases, such as dementia. Thus, in addition to the ventricles, the hippocampus was probed in the present study (Figure 1f). In the hippocampal ROI, MRI signal intensities reached their maxima at 83, 97, and 119 min after the onset of the infusion at rates of 2.92, 1.67, and 1.17 μL/min, respectively. The correlation coefficient was *R* ^2^ = 0.88 (Figure 2e1). Unlike in the ventricles, less contrast agent went into and remained in the hippocampal tissue; thus, the MRI signal enhancement increases were only 8.3%, 7.3%, and 4.0% at peak relative to baseline, respectively. Correlation analysis gave *R* ^2^ = 0.73 (Figure 2e2).

**Figure 3:**
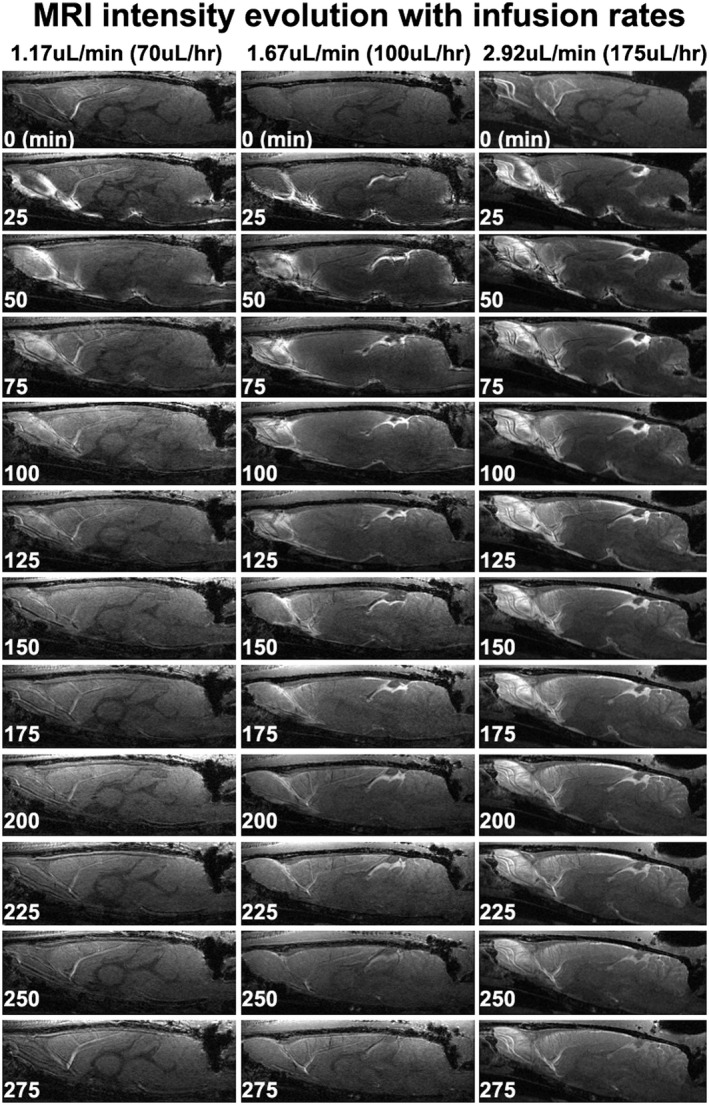
Representative image trains of central sagittal slice of rat brain dynamically acquired by 3D MRI for each group with infusion rates of 1.17 μL/min (left column), 1.67 μL/min (middle column) and 2.92 μL/min (right column). In olfactory bulb, image enhancement faded after 150 min or 200 min post the infusion at rates of 1.17μL/min or 1.67 μL/min, but not for 2.92 μL/min even after 275 min. The rate 2.92 μL/min also caused high magnevist concentration (black holes between the fourth ventricle and cisterna magna) until 100 min after infusion, while 1.17 μL/min and 1.67 μL/min rates resulted in very small black holes at 25 min after infusion

To overview the patterns of MRI image enhancement from influx of magnevist to contrast being cleared out of brain, Figure 3 shows dynamic image trains of the central sagittal slice from representative rat brains acquired by 3D MRI for each group with the different infusion rates. As seen in Figure 3, the olfactory bulb appears to be the most sensitive brain tissue for observing the effect of image enhancement by contrast agent in rat. With a low infusion rate, which equals low dose infused, the image train (left column of Figure 3) demonstrated that the image enhancement visually disappeared after 150 min post magnevist infusion. While magnevist enhancement of MRI images in the olfactory bulb faded 200 min after infusion at the medium infusion rate (medium column of Figure 3). As the fast rate with high dose infusion, contrast enhancement on images can still be easily observed in the olfactory bulb even at 275 min after the initiation of infusion (right column of Figure 3). We noticed that the image trains also demonstrated the T_2_^*^ effect of high magnevist concentration on the MRI 3D gradient echo images, which was apparent as black holes in Figure 3 located between the cisterna magna and the small cavity of the fourth ventricle at early time after infusion. The black holes remained bigger and lasted longer under condition of fast rate (high dose) infusion (right column of Figure 3), and disappeared when the regional magnevist concentration was low enough. The decrease in the regional magnevist concentration is likely caused by combination of completion of infusion, cerebrospinal fluid flow and the brain molecular waste clearance pathway.

**Figure 4:**
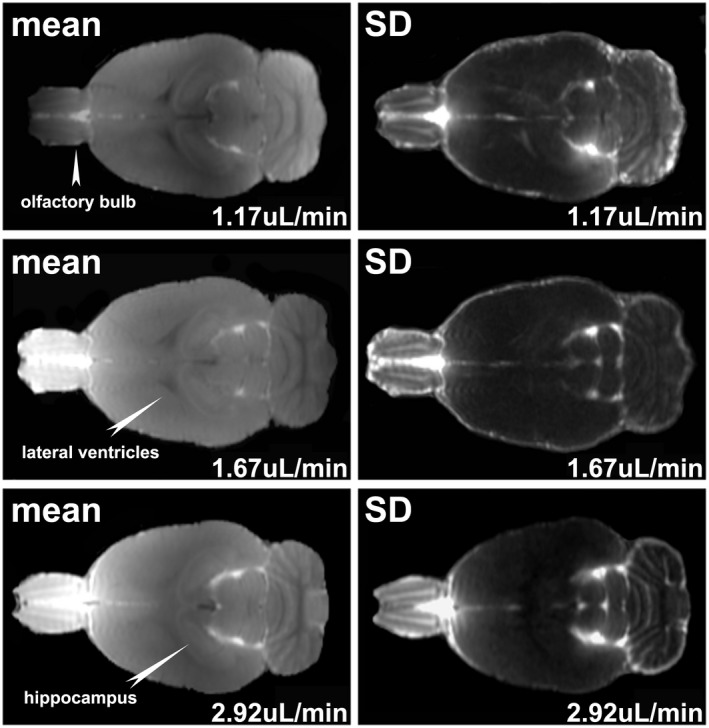
An overview of synthetic MRI intensity changes from representative rats. The mean maps show single coronal slice that represented the individual MRI intensity average of all 55 time points acquired in 5 hr after onset of Magnevist infusion. The *SD* maps demonstrate the corresponding standard deviation maps of the all these 55 images of the same slices for each rat

Figure 4 provides an overview of synthetic MRI intensity changes from representative rats. The left column shows mean maps of a single coronal slice that represents the individual MRI intensity average of all 55 time points acquired in 5 hr after onset of Magnevist infusion. The right column demonstrates the corresponding standard deviation maps of the all these 55 images of the same slices for each rat.

## 4 DISCUSSION

We demonstrated that MRI signal profiles of all ROIs increase quicker and/or higher for fast infusion of contrast agent into CSF, than for slow infusion of contrast agent into CSF (Figure 1). This may be attributed to the relatively low clearance rate of the glymphatic system compared to the influx rate of magnevist. The maximal increase in these profiles depends on the influx rate (related to infusion rate) of contrast agent and clearance rate of glymphatic system. We also have demonstrated that an infusion rate of 1.67 μL/min, that is, 100 μL/hr, is suitable for the glymphatic system study in rat, which provides high increase in image intensity without significant influence on regular CSF flow. This rate has been successfully applied to the study of the glymphatic system in type 2 diabetic rats (Jiang et al., 2016). According to a two‐compartment model of glymphatic transport, (Lee et al., 2015) an increase in infusion rate should only trigger a linear increase in the contrast agent distribution in certain cerebral tissue. However, a high infusion rate is linked to a different pattern of contrast agent distribution than a low infusion rate, since the high infusion rate changes the input function by severely disturbing regular CSF flow.

In the present MRI study of the glymphatic system, by repeating a 3D fast low angle shot sequence without altering any sequence parameters, MRI signal intensity from ROIs of the rat brain should vary and depend only on the magnevist concentration present in the cerebral tissue ROIs after infusion. Magnevist reduces the *T_1_* relaxation time of the cerebral tissue where magnevist accumulated, leading to an increase in steady state magnetization, and thus elevates MRI image intensity acquired with the identical sequence. Because the change of *T_1_* relaxation rate under the condition of brain tissue is proportional to the magnevist concentration, (Tofts, 2010) the evolution of MRI image enhancement of cerebral tissue equally reflects the evolution of magnevist concentration in the corresponding cerebral tissue throughout the MRI experimental scans. Therefore, via changes of image enhancement, the 3D image train of MRI can visibly in vivo demonstrate the influx of molecular magnevist into cerebral tissue and the removal process of molecular magnevist from brain tissue by the glymphatic system in brain.

The increase rate of infusion of magnevist into CSF evokes an increase MRI signal‐to‐noise ratio (SNR) of cerebral tissue to better detect impairment of glymphatic system and provide more reliable data for quantitative analysis. However, it costs more time to observe the entire clearance process. Contrast agent remains in the hippocampus for approximately 5 hr after starting infusion for rates at 1.67 and 2.92 μL/min (3.14% and 4.77% intensity increases above baseline, respectively) of diluted 21.7 mM magnevist in young healthy rats (Figure 1f), and likely it would be even longer in rats with neurological diseases (Jiang et al., 2016) Short times for performance of MRI studies of glymphatic system are required not only for experimental study, but also for clinical application (Ringstad, Vatnehol, & Eide, 2017). Thus, a conflicting circumstance occurs at requiring high dose of tracer for better SNR while limiting time required for continuous observation of glymphatic clearance process.

A fast infusion rate may partly reduce the entire observation time by shortening mangevist infusion time. But there also exists restriction of using high infusion rate of magnevist into the cerebral ventricle in the MRI study of the glymphatic system. Infusion of fluid into CSF may increase the intracranial pressure (ICP) and disturb the regular CSF flow, which may affect glymphatic influx and efflux patterns, and lead to pathophysiological complications and erroneous information. Previous studies demonstrated that an increase in ICP of about 2.5 mmHg, a 50% increase from the baseline of 5.0 mmHg, was observed with a rate of 2.0μL/min infusion in mice, although it did not cause reflux of subarachnoid CSF back into the ventricular CSF compartments, which suggested that the physiological direction of CSF bulk flow is maintained. (Kress et al., 2014) In rat, ICP was slightly increased, approximately 6.3% from baseline of 4.8–5.1 mmHg with the infusion rate of 3.0 μL/min, and no ICP changes were observed with an infusion rate of 0.34 μL/min (Bedussi, Wel, et al., 2017). However, the lowest infusion rate employed in the present study (1.17 μL/min), although lower than 3.0 μL/min, is much higher than the basal infusion rate of 0.34 μL/min, which implies that the infusion may interfere with the physiological flow of CSF, since even small differences in intracranial pressure associated with these infusion rates may induce substantial changes in flow when resistance to flow is low.

Consequently, selection of a magnevist infusion rate is important and must to be tested for MRI study of the glymphatic system. Three relative fast infusion rates (>0.34 μL/min) of 1.17, 1.67 and 2.92 μL/min were selected in the present MRI study of glymphatic system. These rates were from slightly lower (1.17 μL/min) or higher (1.67 μL/min) than half of CSF production rate (2.75 μL/min), to slightly higher (2.92 μL/min) than CSF production rate. Our present MRI results (right column in Figure 3) indicated that the infusion rate of 2.92 μL/min, above the CSF rate of 2.75 μL/min, may induce some retrograde flow, although infusion rate of 2.92 μL/min was slightly slower than 3.0 μL/min which causes less than 6.3% ICP increase (Bedussi, Wel, et al., 2017). The infusion rates of 1.17 and 1.67 μL/min induced only minor Magnevist accumulation around the cisterna magna, but distant from the fourth ventricle shortly after infusion (left columns in Figure 3). Thus, these rates should not have a significant influence on the regular CSF circulation of brain. As noted, we demonstrated that higher infusion rate of magnevist induced increased image enhancement in brain tissue, but took increased time for the brain to clear out magnevist molecules (Figure 3). For example, in hippocampus, increases in MRI intensity remained 4.77%, 3.14% and 0.21% above the baseline at 270 min post magnevist infusion and the maximum increases of intensity were approximately 8.3%, 7.3% and 4.0% from baseline for the twenty minutes infusions at rates of 2.92, 1.67, and 1.17 μL/min, respectively.

Although it has been experimentally and successfully confirmed in a fluorescent imaging study, (Bedussi, Wel, et al., 2017) the infusion rate of 0.34 μL/min is too slow for an in vivo MRI study, For instance, the infusion rate of 0.34 μL/min would require more than 4 hr only for infusion of contrast agent into the CSF in a T2DM study of rat, where 85 μL magnevist was infused (Jiang et al., 2016). Furthermore, there exists competition between the influx of infused magnevist and the clearance mechanism of the glymphatic system. If the infusion rate is too slow, MRI data may not reliably detect and quantitatively analyze image changes of cerebral tissue after magnevist infusion due to large background noise effects and very low accumulated magnevist concentration in brain tissue, especially for a healthy glymphatic system. In addition, concentration of infused tracer is much lower in brain tissue compared with in ventricles, but brain parenchymal response to the CSF infusion is more important for investigation of brain diseases. For instance, the hippocampus plays an important role in spatial navigation and long‐term memory. In hippocampal tissue of rats, MRI intensity increased 7.3% at peak with a 1.67 μL/min infusion rate, and close to a 8.3% increase with 2.92 μL/min infusion rate, and higher than 4.0% increase with 1.17 μL/min infusion rate (Figure 1f). These data indicated that the infusion rate of 1.17 μL/min, with lower image enhancement, is less sensitive to variation of glymphatic system in brain parenchyma.

As is well known and also demonstrated in present study, bound magnevist particles simultaneously have both *T_1_* and T_2_^*^ effects on MRI signal of the hosted cerebral tissue. Accumulated magnevist particles reduced relaxation time *T_1_* of brain tissue, which cause image enhancement with a short repetition time sequence; while the accumulated magnevist particles reduced relaxation time T_2_^*^ of brain tissue, which cause a loss of image intensity. Accumulated magnevist visually caused significantly shorter T_2_^*^ and formed a dark spot near cisterna magna in the 3D MRI images (Figure 3), more evident with the 2.92 μL/min infusion rate (right column in Figure 3 at 25 min). Thus, infusion rate of 2.92 μL/min, surpassing CSF production rate of 2.75 μL/min in rat, is imperfect for the MRI study of glymphatic system in rat. In more precise studies of quantitative mapping or evaluation of glymphatic system by employing MRI, separation of *T_1_* and T_2_^*^ effects, one positive and another negative, may be required (Lee et al., 2018). Using a dual echo sequence is a strategy to primarily separate *T_1_* effect from T_2_^*^ effect in MRI signal (Ewing & Bagher‐Ebadian, 2013). After correcting the T_2_^*^ effect, contrast enhanced MRI may provide more accurate evaluation of glymphatic system.

Neurodegenerative diseases affect clearance efficiency of the glymphatic system in brain parenchyma, which may provide a new way to early diagnose and possibly treat neurodegenerative diseases. Glymphatic function is depressed by brain injury and diseases, such as traumatic brain injury, (Iliff et al., 2014; Plog et al., 2015) stroke, (Arbel‐Ornath et al., 2013; Gaberel et al., 2014) Alzheimer's disease, (Iliff et al., 2013; Taoka et al., 2017) and diabetes (Jiang et al., 2016). The waste clearance was slowed down in impaired glymphatic system with brain injury/diseases, which will necessitate an even longer experimental time than subjects without brain diseases if we observe the whole process of glymphatic clearance. To reduce time required, we may identify impaired glymphatic system by observing partial profiles of glymphatic response to magnevist influx (Figure 1b–f). For example, after 50 min infusion of magnevist at a rate of 1.67 μL/min (100 μL/hr), hippocampal image enhancement reached maximum of 14.5% above baseline at 90 min in healthy rat brain and 27.8% at 155 min in diabetic rats (Jiang et al., 2016). For analysis of a specific part of brain parenchyma or for various neurological diseases, sufficient tracer concentration in cerebral tissue can ensure high SNR and reliable results, and can be achieved by altering the infusion duration with the selected infusion rate.

The present study provides a proof‐of‐principle that MRI can be employed to visualize and evaluate aspects of the glymphatic system. The complexity of this MRI study, including animal surgery, narrow range for selection of reasonable infusion rate, sensitivity of glymphatic response to infusion rate difference, and time consuming to acquire and process MRI restricted our animal populations of this study. Although we successfully demonstrated a dependence of response of glymphatic system on infusion rate in physiologically intact animal, further MRI studies of the magnevist infusion rate on identification of glymphatic and CSF dynamics under pathological conditions are warranted.

In summary, contrast‐enhanced MRI is a powerful tool which provides entire pictures of the working process of the whole glymphatic system in rat brain. After considerations of issues of promoting *T_1_* effect (image enhancement) and depressing T_2_^*^ effect (image reduction) induced by magnevist infusion into CSF, the infusion rate of 1.67 μL/min (100 μL/hr) was found to be suitable in the MRI study of the glymphatic system in adult male Wistar rat. As a caveat, we do not exclude the possibility that the 20 min infusion at rate of 1.67 μL/min may interfere with the physiological flow of CSF in rat brain.

## CONFLICT OF INTEREST

No conflicts are declared for all authors. The content is solely the responsibility of the authors and does not necessary represent the official view of the National Institute of Health.

## AUTHOR CONTRIBUTIONS

*Conceptualization:* MC, ZZ and QJ

*Investigation:* GD, LZ, and QJ

*Data curation:* GD, LL, LZ, ED‐B, QL

*Writing – Original Drafting:* GD

*Writing – Review and Editing: *MC and QJ

## DATA ACCESSIBILITY

To our knowledge, we provide sufficient data in our manuscript (in text and figures) to fully support our results and conclusions. We will fully respond to any request from anyone interested in receiving additional details regarding all experiments and data in our manuscript. However, data are available only for authorized researchers who meet the criteria for access, due to the firewall and our hospital policy.
